# Perceived movement of nonrigid motion patterns

**DOI:** 10.1093/pnasnexus/pgac088

**Published:** 2022-06-22

**Authors:** Krischan Koerfer, Markus Lappe

**Affiliations:** Institute for Psychology and Otto Creutzfeldt Center for Cognitive and Behavioral Neuroscience, University of Münster, Fliednerstr. 21, 48149 Münster, Germany; Institute for Psychology and Otto Creutzfeldt Center for Cognitive and Behavioral Neuroscience, University of Münster, Fliednerstr. 21, 48149 Münster, Germany

**Keywords:** nonrigid motion, visual perception, visual motion field, higher-order motion, curl

## Abstract

Nonrigid materials such as liquids or smoke deform over time. Little is known about the visual perception of nonrigid motion other than that many motion cues associated with rigid motion perception are not reliable for nonrigid motion. Nonrigid motion patterns lack clear borders and their movement can be inconsistent with the motion of their parts. We developed a novel stimulus that creates a nonrigid vortex motion pattern in a random dot distribution and decouples the movement of the vortex from the first-order motion of the dots. We presented three moving vortices that entailed consecutively fewer motion cues, eliminating occlusion, motion borders, and velocity field gradients in the process. Subjects were well able to report the end position and travel path in all cases, showing that nonrigid motion is perceived through an analysis of the temporal evolution of visual motion patterns and does not require borders or speed differences. Adding a coherent global motion did not hamper perception, but adding local noise did, indicating that the visual system uses mid-level features that are on a local scale. We also found that participants judged the movement of the nonrigid motion patterns slower than a rigid control, revealing that speed perception was based on a combination of motion of the parts and movement of the pattern. We propose that the visual system uses the temporal evolution of a motion pattern for the perception of nonrigid motion and suggest a plausible mechanism based on the curl of the motion field.

Significance Statement The visual perception of the nonrigid motion of liquids, smoke, or fire is complicated since these motion patterns lack many motion cues known to be used for motion perception. We developed a novel type of stimuli that can decouple the movement of nonrigid motion patterns from the motion of their parts. Using these stimuli, we show that patterns in the visual motion field can be detected and furthermore that the change of the motion field over time is perceived as the motion of an entity—a so far unreported type of higher-order motion percept. We further show that the relevant information is extracted on a local scale and propose a plausible novel perceptual mechanism based on consecutive local derivatives.

## Introduction

Ocean waves, camp fire, undulating fields in the wind, and flocks of birds—deformable patterns of nonrigid motion provide a special pleasure to the eye. Such motion patterns can capture the attention of our visual system for a long time, presumably because they keep changing while also keeping the same. Yet, the ability to understand nonrigid motion is also important for survival: think of raging fires, storm clouds, water vortices, and crowds of people in flight. While introspection suggests that humans are able to perceive nonrigid motion, the extent, the requirements, the limitations, and the perceptual mechanism of this ability await deeper study. Prior research on visual motion has uncovered intricate details of the perception of the motion of single rigid objects (1), the patterns of optic flow generated by self-motion through a rigid visual scene (2–4), and the patterns of biological motion generated from the actions of other humans or animals (5–9). These perceptual processes rely on fixed physical relationships between the parts that make up the object, scene, or animal (5,10–14).

Nonrigid motion is different. First, the parts are not in a fixed relation to each other but move individually, The resulting visual motion pattern may undergo deformations over time. Second, the parts that compose such a visual motion pattern can change over time, too. Belongings and borders are usually not well-defined: birds may join or leave the flock, water molecules may be dragged into a vortex or spilled out. Third, the visual motion pattern itself may move. Such movement of the entire pattern is not bound to the motion of the parts, but rather forms an independent emerging phenomenon. For example, a water vortex may travel down a river while the water molecules in the vortex move around its center, or a wave travels across the ocean surface while the water droplets just move up and down. Fourth, unlike for rigid-motion, the speed and direction of the movement of a nonrigid visual motion pattern is independent from the speed and direction of its parts: a fast rotating water vortex can be stationary while a slow rotating water vortex can travel downriver quite fast. Fifth, since the visual motion pattern is defined by the motion of its parts (rather than by other properties of the parts, like being a bird or being close to other birds) there can be no occlusion. When a gust of wind causes a wave to travel across a wheat field, the traveling wave never occludes the wheat. Instead, the wheat becomes part of the wave pattern for a short time as one wheat row after another bends and then unbends again. Sixth, there is often no sharp kinetic boundary for nonrigid motion patterns. While the motion signals of moving rigid objects always contain a boundary between the object and its surrounding, this is not the case for nonrigid motion. A water vortex, for example, has a smooth transition of slow and fast moving water without any boundary.

**Fig. 1.**
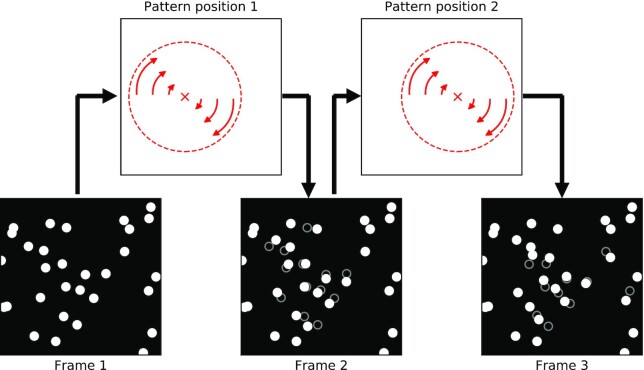
Stimulus construction for a nonrigid visual motion pattern moving through a field of dots. The stimulus consists of randomly distributed pixel-sized white dots on a black background (lower row). Between frames the dots move according to a vortex pattern shown in the upper row. For illustration, frames 2 and 3 show the new dot positions (filled dots) along with each dots former position in the preceding frame (open circles). In addition, the center of the vortex shifts with each frame (upper row). Thus, in each frame the center position around which the dots rotate is in a different location. The resulting movement of the vortex pattern features the properties that set nonrigid motion apart from rigid motion: the change in position of each dot between frames produces a rotating visual motion pattern. The movement of this vortex pattern is independent from the the motion of its parts (the dots), as they do not follow the movement of the vortex between frames but solely rotate around the current center of the vortex. New dots become part of the vortex in the next frame as the vortex travels along, while dots at the trailing part fall out. Thus, the vortex does not consist of a fixed set of dots and there is no occlusion. Importantly, the dot motion field, i.e. the motion of the dots between any two frames, is always that of a stationary vortex. The movement of the vortex is only entailed in the temporal evolution of the motion field over time, i.e. in the way the motion field changes between frames.

We have developed a novel visual motion stimulation that allows to study these properties of nonrigid motion perception in a controlled way. In this stimulus, a visual motion pattern of a vortex is defined by a field of dot motion. The movement of this motion pattern emerges solely from the temporal evolution of the dot motion field and is fully independent from the motion of the dots themselves (Fig. 1). The stimulus consists of white dots placed on a black background. Each dot can be stationary or moving. Speed and direction of a single dot’s motion are governed by the dynamics of a vortex centered at a particular position in the dot field. The vortex moves across the screen by shifting the position of its center from one frame of the animation to the next. Importantly, the dots do not move along with the movement of the vortex center. Instead, in the new frame each dot is assigned a new velocity according to the new vortex center position. Therefore, no dot motion is generated by the movement of the pattern. The motion of the dot field is always that of a stationary vortex, yet the vortex pattern moves across the screen over time.

This stimulus features all the properties that set nonrigid motion apart from rigid motion. Moreover, motion cues that usually accompany both rigid and nonrigid motion are removed in order to investigate the properties intrinsic to nonrigid motion in isolation. In this stimulus, motion perception of the vortex could not be based on luminance, orientation, contrast, occlusion, dot-density, or dot motion since this information was either absent or not coupled to the movement of the vortex. We used this stimulus to investigate whether the remaining information, the movement of the vortex pattern, can be used for nonrigid motion perception and to study the mechanisms of this perceptual capability.

## The Movement of Visual Motion Patterns can be Used as a Motion Cue for Accurate Nonrigid Motion Perception

We first determined which cues are essential to perceive the movement of a vortex pattern. We presented three stimulus variants of the vortex containing successively reduced sets of cues, and one rigid motion control stimulus. In order to investigate how accurate motion perception was depending on available cues, we asked participants to report the final position of the vortex center and its travel path.

**Fig. 2.**
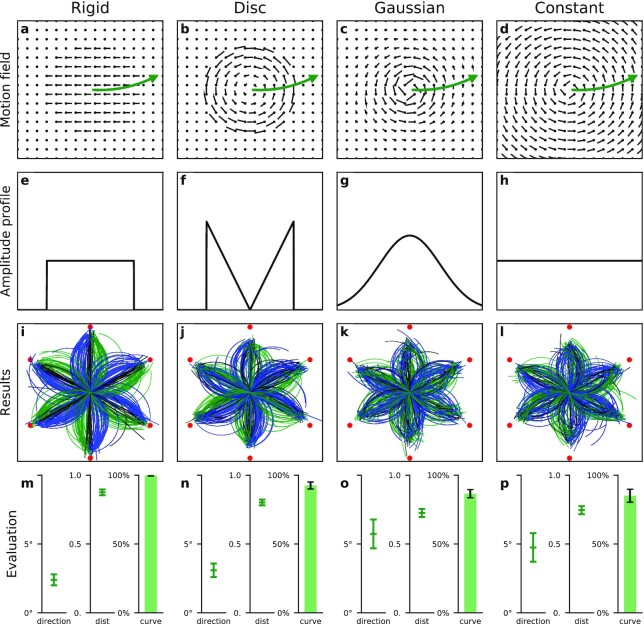
Stimuli and results of the motion tracking experiment. Top row depicts the dot motion fields for four stimuli containing different cues to nonrigid motion perception (Table 1). In each trial of the experiment, the center positions of the motion patterns moved (illustrated by the green arrows). Participants had to report the perceived path of movement. Second row shows the spatial profile of the dot motion speeds. Rigid control and Disc stimuli feature sharp motion boundaries, Gaussian a smooth transition and Constant no change in dot speed anywhere. Third row illustrates the task and shows the participants’ responses. The vortices moved along a curved path (green arrow in top row) from the middle of the screen toward one of six possible end-positions (indicated by red dots). Each line in panels (i)–(l) shows the reported end-position and movement path of a single trial by a single participant. Lines are colored based on the curvature of the true movement path: blue for clockwise, green for counterclockwise, and black for straight. The bottom row shows the quantitative analysis of participants performance: direction: the mean absolute error in direction of the reported end-position in degree; dist: the mean fraction of reported and real distance of the movement of the vortex; and curve: the percentage of correctly reported clock- and counterclockwise curvature (not counting straight/black). Error bars indicate 95% CI.

Fig. 2 shows the four stimuli and results arranged in decreasing number of cues. Table 1 lists the cues contained in the different stimuli.

**Table 1.** Available motion cues for the rigid control stimulus and the three nonrigid stimuli.

Motion cue	Rigid	Disc	Gaussian	Constant
Dot motion in the direction	$\checkmark$	x	x	x
of the movement of the pattern				
Occlusion	$\checkmark$	x	x	x
Edge / kinetic border	$\checkmark$	$\checkmark$	x	x
Dot motion gradient	($\checkmark$)	$\checkmark$	$\checkmark$	x
Moving pattern in the dot motion field	$\checkmark$	$\checkmark$	$\checkmark$	$\checkmark$

The Rigid control condition (Fig. 2a) presented the motion of a solid nonrotating object, a circular plate that moved across the screen, and contained all cues available for rigid motion. The motion of each dot was bound to the motion of the plate as a whole. Dots were either part of the plate or part of the background and kept their assignment throughout the plate’s movement, featuring occlusion and a sharp kinetic boundary. The plate was initially presented in the center of the dot field and then moved rigidly along a slightly curved path toward one of six eccentric positions (red dots in third row of Fig. 2). Participants were asked to redraw the perceived path of the object. They first clicked on the perceived final position of the center of the object. Then, a curved line connecting the start- and perceived end-position appeared. Participants had to drag the mouse to alter the curvature via the segment height and click again once the line matched the perceived curvature of the movement path. Thereafter, the next trial started. Figure 2(i) shows the results. Each of the colored lines depicts a single trial of a single participant. The colors indicate the direction of the original path curvature (blue: clockwise, green: counterclockwise, and black: straight path). The reported end positions are close to the correct end positions (red dots) of the plate center and all reported curvatures matched the direction of the original curvature. To analyze the participants’ performance quantitatively we determined three performance measures: the mean absolute error of direction (direction error), the mean absolute error of distance (distance error), and the proportion of trials in which a curvature was reported in the wrong direction (curvature error). These errors were determined for each participant and stimulus type. The direction, distance, and curvature errors confirm high performance for the Rigid control stimulus (Fig. 2m). The performance on the stimulus presenting rigid motion of a solid object and containing many cues about the motion and location of the plate (Table 1) is the baseline of our comparative analysis. The next stimuli presented nonrigid motion and systematically reduced the number of available cues.

The Disc condition (Fig. 2b) presented a vortex with a sharp border. Dots within the vortex borders rotated around the center with speed increasing linearly with distance from the center. Dots outside the borders of the vortex were static. Between animation frames the center of the vortex shifted, causing the vortex to move across the screen. In each frame, some formerly static dots became part of the vortex while others left the vortex, such that no individual dot followed the vortex movement and the vortex movement was independent of the dot motion and not linked to any fixed subset of dots. Thus, occlusion and dot motion in direction of the vortex’s movement that were present in the rigid motion were eradicated (Table 1). The results (Fig. 2j and n) show that perception of the vortex movement was still accurate. Mean direction error was small and direction of curvature was accurately reported. However, the perceived distance that the center of the vortex moved was somewhat underestimated. This may be due to one of the essential differences between the Rigid control and the Disc stimulus. The rigid-motion control stimulus contained overall dot motion in the direction of the plate’s movement, which the nonrigid Disc stimulus lacked. We will come back to this in a further experiment below. Yet, despite this difference, the results for the Disc stimulus show that nonrigid motion perception does not collapse without occlusion or dot motion in the direction of the movement of the vortex, the two most prominent cues of rigid object motion. However, the perception of the location and travel path of the vortex in the Disc condition might be supported by a kinetic boundary that could be used to segment the vortex. Tracking of the segmented vortex over time could then be used for travel path perception.

The Gaussian condition (Fig. 2c) removed the kinetic boundary. In this stimulus, the rotational speed was high at the center and slowed down gradually toward the periphery, similar to a natural water vortex. The speed of the dots followed a Gaussian profile centered at the center of the vortex. Thus, the stimulus contained a speed gradient and a salient center for tracking but no kinetic boundary or sharp motion edge that separated the vortex from the background (Fig. 2g and Table 1) Yet, perception of the travel path of the vortex was again quite accurate (Fig. 2k and o), demonstrating that motion perception was still possible. A slight decrease in performance compared to the Disc condition indicates that kinetic boundaries may be helpful but are not essential for nonrigid motion perception.

Finally and crucially, the Constant condition removed all information about the vortex movement from the velocities of the dot motion field. Dots rotated around a center with a speed that was constant throughout the field. The only cue left was the movement of the vortex pattern, i.e. the temporal evolution of the dot motion field. At any moment in time, the dot motion field described the motion of the dots at each position as a rotating vortex at a particular location. The movement of the vortex, in contrast, was contained in the way the dot motion vectors change over time. Determination of the vortex movement, thus involves a second time derivative on top of the spatio-temporal derivative that is required to determine the motion of each dot from the local luminance changes (so-called first-order motion). Importantly, the second derivative required to determine the movement of the vortex is not the same as the second derivative that would be needed to measure acceleration of a dot. A dot motion field can contain areas where dots accelerate while the field itself does not change over time, and dot acceleration is not tied to the movement of the vortex pattern. In case of a nonmoving vortex for example, each dot is continuously accelerated perpendicular to its motion direction, because of to the rotation, but the motion field itself is static in time. In the Constant condition, participants succeeded in detecting the motion of the pattern even as the temporal evolution of the dot motion field was the only cue to solving the task (Fig. 2i and p). Performance with regard to direction, distance, and curvature errors was at the same level as for the Gaussian condition. We conclude that speed differences in the dot motion field, which were present in the Gaussian condition, are not essential for nonrigid motion perception. Instead, our results show that in addition to other established motion mechanisms the human visual system can also use the movement of a visual motion pattern, i.e. its temporal evolution, as a cue for nonrigid motion perception, since this was the only cue present in all conditions.

## Patterns of Visual Motion are Processed on a Local Scale

Our results so far have established that the movement of a visual motion pattern over time is a crucial cue for the perception of nonrigid motion. Neural mechanisms for the perception of visual motion patterns, including rotation, have been described in higher areas of the motion pathway in the primate brain (15–18). These neurons typically respond best to large patterns. Their sensitivity is regarded as a form of complex global pattern matching. If a mechanism of global pattern matching is involved also in the perception of our vortex stimuli one might expect a model that proceeds as follows. It starts with the motion field of all local motion vectors. Then, a global pattern matching across the entire field reveals the position of the vortex center at the current time step. Third, a spatio-temporal analysis of the center position derives the movement of the pattern. Alternatively, the mechanism could not use global but local information of the motion field. The use of local temporal derivatives of the visual motion field has been proposed long ago for the perception of rigid motion and heading of self-motion (19–21). However, previous research investigating the use of local derivates in human heading perception found no support (3). Investigations of flow sensitive neurons in area MST of the primate brain showed that the corresponding receptive fields were too large and, importantly, that global translation (i.e. the global disturbance in our study) significantly degraded the response of these neurons (22). These are compelling arguments against the use of local derivatives in heading perception from optic flow. However, using local derivatives of the visual motion field might be more useful for nonrigid motion perception when global correspondences are not available. This is usually the case in natural nonrigid cases such as turbulent flow, smoke, or fire. In our experiments, the main first-order derivative of relevance is the curl of the dot motion field. A model based on local derivatives for detecting the vortex motion might proceed like this: it also starts from the motion field of all local motion vectors. Next, however, local derivatives in small neighborhoods are computed to extract the curl of the motion field at any field position as mid-level feature. Third, a spatio-temporal analysis of the maxima of the curl field reveals the movement of the pattern. Hence, the two models differ only at the mid-level stage. All visual motion patterns used in the four stimuli cause a unique curl field with maxima at the dynamic key points that could allow localization and tracking (Figure S1, Supplementary Material). The displacement of the dynamic key points in the curl field over time could then be used to track the travel paths.

**Fig. 3.**
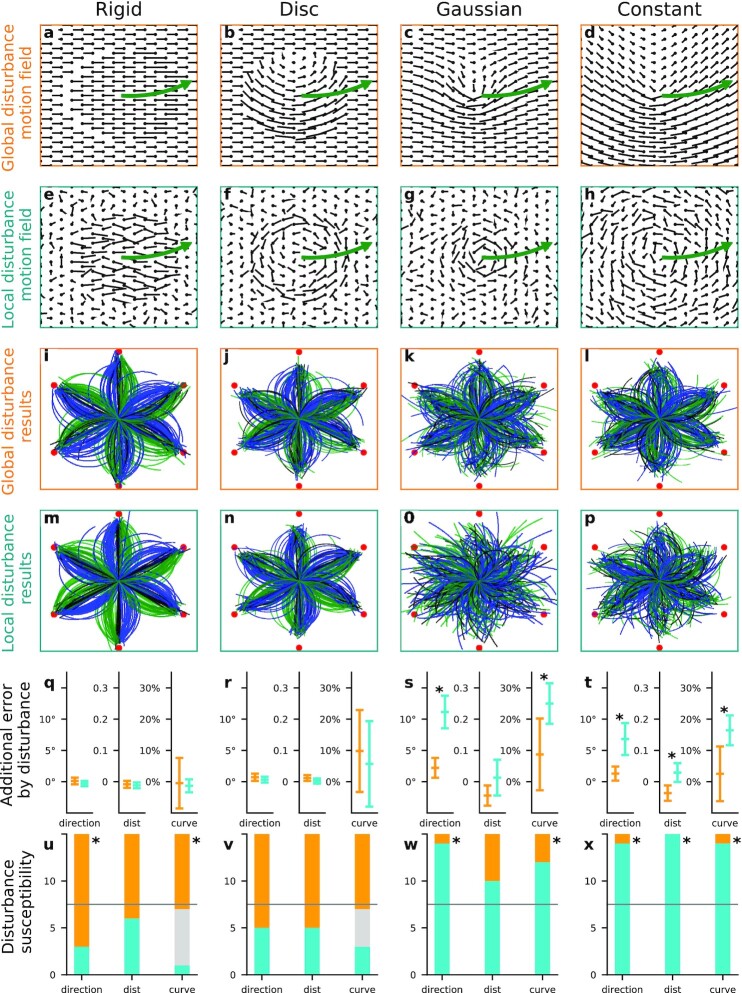
Effects of global and local disturbances on nonrigid motion perception. Top row: when a common sideways motion is added to all dots the global pattern does not resemble a vortex rotation anymore. Second row: adding random noise to each individual dot motion retains the global pattern but disturbs relative motion between neighboring dots. Third and fourth row: responses of the participants in the different conditions. Fifth row: additional direction, distance, and curvature errors introduced by the global (orange) and local (blue) disturbances. Additional error is the difference between the error in the disturbed condition and the error in the undisturbed condition. Also shown are the corresponding 95% CI. Sixth row: disturbance susceptibility indicates how many participants were more affected by either the global (orange) or the local (blue) disturbance condition in regard to perception of direction, distance, and curvature. Asterisks in row five and six indicate a significant difference between global and local disturbance condition.

To distinguish between these two models, i.e. the global pattern matching model or the local derivative model, we created two disturbance conditions designed to affect the stimulus either on a local or on a global scale and specifically disturb one model or the other. The global disturbance condition added a uniform leftward or rightward motion component to all dots in the stimulus (80% of the speed of the movement of the motion patterns). When this motion is added to the dot motion field the resulting visual motion patterns do not globally resemble vortices anymore (Fig. 3b–d). This severely disturbs the global pattern matching but not the local derivatives. At the local scale, the relative motions of neighboring dots with regard to each other are the same as in the regular vortex pattern, since the addition of a common sideward motion to all dots does not change the differences in motion between neighboring dots. Hence, detection based on relative motion at a local scale, as is done in the analysis of local derivatives, should be unaffected.

Conversely, the local disturbance condition (Fig. 3e–h), added small motion vectors of random direction and magnitude to each individual dot. The average speed was equal to the speed of the sideward motion in the global disturbance condition. The local disturbance condition introduced disturbance on a local scale that was not correlated across the field. Thus, the relative motion between neighboring dots changed and local derivatives would be affected strongly. The impact on global pattern matching should be small since the small random additions should cancel out if integrated across the large field and the pattern still globally resembles a (noisy) vortex (Fig. 3e–h).

The results showed for the Rigid control stimulus a mostly unaffected performance in either disturbance condition (Fig. 3i and m) when compared to the unperturbed stimulus in Fig. 2(i). For each participant, we also compared their performance in the two disturbance conditions with their prior undisturbed performance quantitatively. First, for each participant, stimulus type and disturbance condition we determined three performance measures: the mean absolute error of direction, the mean absolute error of distance, and the proportion of trials in which a curvature was reported in the wrong direction. Second, for each participant, the difference of these errors between the undisturbed condition and the local/global disturbance condition was calculated. These differences are named additional direction, distance, and curvature errors from here on. Figure 3(q)–(t) shows the means of the additional errors across all 15 participants and the corresponding 95% CI. For the Rigid condition, there is no significant impact on performance, so perception of the rigid control stimulus was very robust versus both disturbances. Performance for the Disc stimulus (Fig. 3j and n) appeared likewise unaffected in comparison to Fig. 2(j). The additional errors shown in Fig. 3(r) confirm this with no significant drop in performance, just the CI for the additional curvature error are larger, which was caused by a higher variance in participants’ performance for the Disk than for the Rigid stimuli. Thus, neither of the disturbances was strong enough to interfere with perception when many motion cues were available in the Rigid control and Disc stimulus. The Gaussian and Constant condition, in contrast, showed a clear susceptibility to local disturbances (Fig. 3o and p). The response patterns became incoherent, indicating a degradation of perception. The global disturbance, in contrast, gave response patterns (Fig. 3k and l) that were similar to those of the undisturbed stimuli (Fig. 2k and l). That the local disturbance degraded perception for the Gaussian and Constant stimulus can also be seen in the quantitative analysis in Fig. 2(s) and (t). For the Gaussian stimulus the additional direction error was 11.1° ± 2.6° for the local disturbance, which was significantly more than the additional error by the global disturbance (paired t test, *t* = 6.50). Participants also reported additional 25% ± 6% of the curvatures in the wrong direction in the local disturbance condition compared to the undisturbed condition. This increase in error was significantly higher than for the global disturbance (paired t test, *t* = 3.77). For the Constant stimulus, the local disturbance induced additional error of 6.8° ± 2.5° in direction, 0.29 ± 0.03 in distance, and 16.4% ± 4.8% in curvature. These were all significantly higher additional errors than the corresponding additional errors caused by the global disturbance (paired t test, *t* = 4.48, *t* = 5.17, and *t* = 3.12).

To analyze the influence of local and global disturbances for the different visual motion patterns deeper, we also determined for each condition whether participants were more affected by the local or the global disturbance (Fig. 3q–t). For each single participant, it was checked whether their mean performance over all trials within one pattern condition was worse in the global or the local disturbance condition with regard to direction, distance, and curvature errors. For the Rigid control and the Disc stimulus, participants tended to be affected slightly stronger by the global than by the local disturbance. The yellow bars in panels (q) and (r) of Fig. 3 show the number of participants that were more affected by global disturbance. Blue bars give the number of participants that were more affected by local disturbance. Gray bars indicate the number of participants that were equally affected by local and global disturbance. This happened for the curvature error when participants made the same number of errors in both conditions. Asterisks indicate that a significant number of participants performed worse on the global disturbance condition for direction (*P* = 0.035) and curvature (*P* = 0.039) perception (two-tailed sign test). In contrast, in the Gaussian and Constant conditions most participants were clearly more affected by the local disturbance. For the Gaussian condition participants performed significantly worse with local disturbance for direction (*P* < 0.001) and for curvature (*P* = 0.035). For the Constant condition performance was worse with local disturbance for direction (*P* < 0.001), distance (*P* < 0.001), and curvature (*P* < 0.001). We conclude that motion perception for the Gaussian and Constant condition was more strongly affected by local disturbance. Since the global disturbance affected performance more for the Rigid control stimulus while the local disturbance affected performance more for the Gaussian and Constant stimuli, neither disturbance could be seen as generally stronger or more challenging than the other. Instead their effects on performance depended on which cues were present and how these cues are processed by the visual system. Since the local disturbance but not the global disturbance degraded perception for the Gaussian and Constant stimulus, the data rejects the global pattern matching model and favors the local derivative model.

## Nonrigid Motion Appears Slower than Rigid Motion

In the experiments above, participants underestimated how far the vortices moved across the screen. We wondered whether this underestimation may be linked to a misperception of the speed with which the vortices moved. Speed perception is a key component of motion perception. Complementing the previous experiment of localization and tracking, we therefore, investigated the perception of the travel speed of the Disc, Gaussian, and Constant vortices compared to the Rigid control. In this experiment, two visual motion patterns were successively presented that moved left or right for a random duration. One of them was the Rigid control stimulus and moved at a fixed speed. The other was one of the vortices and moved with different relative speeds in different trials. Participants (*n*= 15) had to report which of the two stimuli appeared to move faster.

**Fig. 4.**
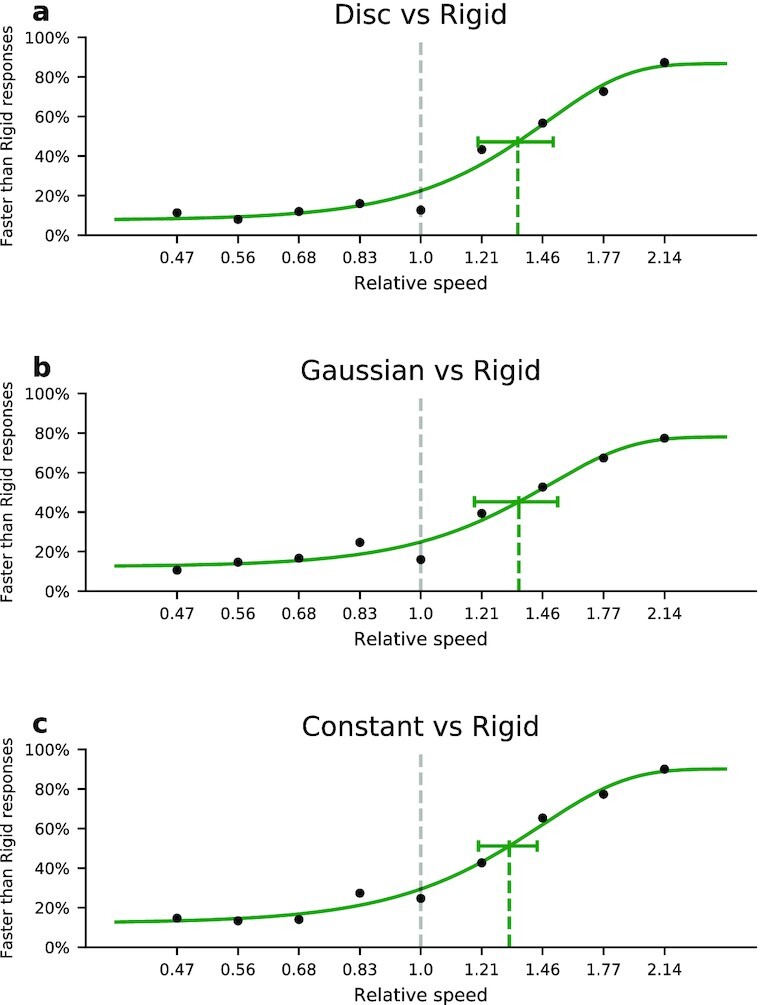
Psychometric curves for the speed discrimination task. Participants indicated whether the movement of one of the nonrigid vortex patterns appeared faster or slower than that of the Rigid control stimulus. Green dashed lines indicate the PSE along with the 95% CI. All PSEs are significantly greater than 1 showing that the movement of the vortex appeared significantly slower than that of the Rigid control stimulus for all conditions. Nonrigid motion was perceived as equally fast as the rigid motion when it was in fact about 35% faster. PSEs were not different between the nonrigid conditions.

We fitted psychometric curves on the accumulated data of all 15 participants for each of the conditions and computed points of subjective equality (PSEs) at which the nonrigid and the rigid motion appeared equally fast (Fig. 4). We found that all nonrigid motions were perceived as significantly slower than the Rigid control motion (two-tailed t tests against a PSE of 1 based on the SE of the least-square estimators for the PSEs, Disc:*t* = 17.08, *P* < 0.001; Gaussian: *t* = 15.45, *P* < 0.001; and Constant: *t* = 21.83, *P* < 0.0001). They were perceived as equally fast when they traveled in fact about 35% faster. There was no significant difference between the nonrigid patterns (ANOVA based on the SE for the least-square estimators of the PSEs: *alpha* = 0.05, *F_c_* = 3.4, *F* = 0.185, and *P* = 0.832).

In the Rigid control stimulus, the movement of the visual motion pattern and the motion of the dots are in the same direction. In the nonrigid stimuli, the motion of the dots is decoupled from the movement of the pattern. Since the dot motion produces zero average motion in the direction of the vortices’ movement, our finding that the speed of the nonrigid object can be perceived at all (albeit too slow) is clear evidence that a distinct mechanism for perception of the speed of nonrigid motion exists. Moreover, since all three nonrigid motion stimuli were perceived as equally fast, the perception must rely on the sole cue that was available in all conditions, namely the movement of the visual motion pattern.

## Discussion

Nonrigid motion is a complex conglomerate of motions of different scale and quality. The two most prominent motion types are the motions of parts (a water droplet, a spark, and a bird) and the movement of motion patterns of the parts (vortices and waves). The motions of the parts are only casually coupled to the pattern motion since they can be of different, dynamically changing direction and magnitude. We investigated how the perception of the movement of a visual motion pattern is derived from the motion of its parts. Using stimuli in which the two motions were fully decoupled and different motion cues could be fully controlled we found that participants evaluated the temporal evolution of the dot motion field to perceive the movement of the pattern.

Perception of the movement of a visual motion pattern is a form of higher-order motion perception. Higher-order motion perception consists of a two-step process. First, information about a certain visual property, a feature, is extracted from a map of the visual scene at each moment in time. Such features can be texture, flicker, and motion boundaries (second-order motion (23–25)), body postures (biological motion (14)), or various other features that can be tracked, e.g. motion modulation (third-order motion (26–28)). In a second step, spatio-temporal filters detect the change of this feature over space and time (posture–space and time for biological motion) and produce the motion percept. A similar mechanism might apply for the movement of the vortex patterns in our study. Then the essential questions becomes which feature is used in the first-step of such a mechanism.

Our results showed that the relevant information for this mechanism is obtained on a local scale, since for the Constant stimulus, which had the movement of a motion pattern as sole cue, distortions added at a local scale produced more perceptual disturbance than distortions added at a global scale. In nature, utilizing the information on a local scale is a prerequisite for most nonrigid motion perception since turbulent motion, for example, cannot be captured on a global scale. For these reasons, it appears likely that the mechanisms for nonrigid motion perception utilize local vector operators on the visual motion field. Usage of the curl of the visual motion field would be especially fitting to the results of our study as the centers of the visual motion patterns correspond to maxima in the curl field. Spatio-temporal filters imposed on a map of curl in the visual motion field would be able to produce the results of our study. A mechanism based on the shear of the motion field would also be activated by the stimuli presented in the study, although to a lesser extent, and is a potential alternative. However, a mechanism based on curl of the motion field (in physics also called vorticity) is the most obvious explanation to the finding that the movement of these vortices can be perceived. Such a model contains three nested derivatives. First, spatio-temporal filters are applied to the temporal evolution of the luminance to construct a motion field describing first-order motion at every position. Second, the curl of this motion field is calculated using the cross-product of spatial derivatives of the motion field. As third step, there are two possibilities. Either spatio-temporal filters are applied directly to the temporal evolution of the curl field, resulting in the motion percept of the vortex. Or some entity is derived from the curl field at each point in time (e.g. its maximum or a center of mass) and tracking of this entity over time results in the motion percept.

The building blocks of these models could have multiple purposes extending beyond motion perception. The curl of the motion field is already a rich information source without considering its temporal evolution. Earlier studies showed that the relations of local motion vectors are the critical feature for apparent liquidness and viscosity (29). As the curl depends on the relations of local motion vectors, these results can be well explained with the visual system using curl for liquidness and viscosity perception.

Moreover, a mechanism of estimating the temporal evolution of local motion field derivatives has not to be limited to curl. Other local derivatives of the motion field like divergence or shear (19–21) might also be useful in analyzing nonrigid motion. In this study, we deliberately constructed the visual motion patterns to be divergence-free since we aimed to remove all cues other than the temporal evolution of the motion pattern. Divergence would inevitably produce other cues in the dot field over time. For example, if there were a sink in the motion field, which equates nonzero divergence, dots would accumulate in the sink producing additional cues of dot density and luminance. These cues would reveal the sink position even in a static image. Such stimuli would not be suitable to investigate the proposed motion mechanism in isolation. While it might be possible to reduce the impact of such cues experimentally by using limited lifetime stimuli, we choose divergent-free vortex stimuli as the most straight forward approach to our main research questions.

Yet, shapes emerging from the orientation and density of parts caused by the parts’ motion are often an integral component of a natural scene containing nonrigid motion. For example, a water vortex is defined by the motion of water droplets, and yet the water droplets tend to also form a shape that is still visible in a photo of the vortex. Thus, even a single static shape might convey information about nonrigid motion, for example to extract the viscosity of liquids, a physical property that is closely tied to motion (30). As such shapes undergo rapid transformations, perception of nonrigid motion based on temporal evolution of shape might also be possible.

Another key result of the study was that the speed with which the nonrigid vortex pattern moved appeared slower than that of the Rigid control. This was true for all three nonrigid stimuli with no significant differences between them. The slow appearance does not seem to rely on a bias for slow motion percepts that is used in Bayesian models when only few cues or unreliable information is available (31,32). If this were the case, one would expect that the Constant pattern would be perceived even slower than the Disc pattern as the former contained fewer motion cues than the latter. This was not observed. Instead, we believe that the reduced speed results from a conflict between the first-order motion of the dots and the higher-order motion of the pattern movement. Higher-order motion is in principle perceived as equally fast as first-order motion (33). However, in our nonrigid stimuli, first-order motion was not absent but merely disconnected from the movement of the vortex. The first-order motion in the nonrigid stimuli, when integrated over the whole display, indicated a net motion of zero. In the Rigid stimulus, in contrast, the first-order motion of the dots of the plate and the movement of the plate were in the same direction. Perhaps the conflicting first-order and higher-order motion in the nonrigid stimuli produced a slower percept than in the nonconflicting Rigid stimulus. Such conflict can reduce perceived speed, as was shown for moving Gabor patches with conflicting internal first-order motion (34). Perceiving the nonrigid visual motion patterns as moving slower is also consistent with perceiving the path of their movement as shorter in our first experiment.

Motion, in nature, is the result of a physical process. The dynamics of motion depend on physical properties interacting with each other governed by the laws of physics. For simple interactions of moving rigid objects some physical properties have an obvious corresponding perceptual dimension: physical velocity is related to motion perception and physical position is related to perceived location. But physical properties that are only indirectly observable, like force, can be perceived and used, too, for example to form the concept of causality (35). This shows that a concept of the dynamics of rigid motion described by Newtonian mechanics is embedded in the visual system (36). For nonrigid motion, the dynamics are much more complex and it is unclear how much of these dynamics are incorporated in the visual system.

Recent studies showed that humans can estimate viscosity of liquids from visual motion, showing accurate perception of a fundamental, but only indirectly visible, physical property (37). Furthermore, fundamental physical properties can be used to predict the motion path of nonrigid cubes after collisions, which necessitate perceiving and utilizing the elasticity of the cubes (38). Both studies imply some understanding of nonrigid physical dynamics on a perceptual level. Our study is concerned with the mechanisms behind the perception of nonrigid physics. We showed that the visual system uses the temporal evolution of the motion field for nonrigid motion perception, rather than the instantaneous motion field or the motion of individual parts. While this study showed that the temporal evolution of the motion field as sole cue is sufficient for motion perception, further research about the importance of this cue and its interaction with the many different cues available in complex real-life nonrigid motion is warranted. The use of the temporal evolution of the motion field might be especially beneficial because it contains more information about the physical dynamics than the instantaneous motion field or the motion of individual parts. For a perceptual concept of physical dynamics, the temporal evolution of the visual motion field might be a crucial information source not only for perception of nonrigid motion but also to detect the forces at work and to predict future states.

## Methods

The study was conducted with 15 participants (nine female and six male) with age ranging from 20 to 36 years, naive to the purpose of the study. The study was approved by the Ethics Committee of the Department of Psychology and Sport Science of the Westfälische Wilhelms Universität, Münster, Germany. Stimuli were created with Python 3.5, SciPy 1.1.0 and Psychopy 3.0 on an Apple MacBook Pro 2017 equipped with a Radeon Pro 560 graphic card with 4,096 MB memory. Stimuli were presented on an Iiyama MS102DT—Vision Master 505 Monitor with a 40 × 30 cm CRT screen with 1,600 × 1,200 pixels and 60 Hz refresh rate. Viewing distance was 1 m, resulting in 69.8 × 52.4° visual angle. Each frame of the stimulus consisted of pixel-sized whites dots with a density of 2.3 per square degree on a black background.

Between frames, dots moved according to different motion patterns (Fig. 2a–d). In the Rigid control condition dots were attached to a black plate with a radius of 7.0° that moved in front of the background, occluding it causing both accretion and deletion boundaries but no density cue. In the Disc condition, the dots rotated around a center with fixed angular speed of 0.6 rad/s, and hence increasing linear speed (Fig. 2f), up to also 7.0° from the center. Dots further away remained static. In the Gaussian condition the rotational speed followed a Gaussian profile with maximal speed at the center (Fig. 2g). The Gaussian function parameters were chosen such that the total integral of dot motion speed was equal to that of the Disc condition and the standard deviation was 7.0°, the edge of the Rigid and the Disc condition. In the Constant condition all dots on the screen rotated around a center with a fixed linear speed (Fig. 2h), hence with decreasing angular speed with distance from center. The speed was set to match the average speed of moving dots in the Disc condition.

For the motion tracking task, the patterns moved with 7.0°/s from the center of the display to six possible end positions. The end positions were 14.0° from the center at an angle of 30°, 90°, 150°, 210°, 270°, and 330° (with vertical up from the center defined as 0°). The patterns traveled on the curved part of a circular segment, with segment heights *h* a factor of −0.2, −0.1, 0, 0.1, or 0.2 of the distance from center to end position. The motion lasted for 2 s and 120 frames. The last (static) frame was shown until participants responded. Participants first had to click on the perceived end-position. Then, a curved line connecting the start- and perceived end-position appeared. Participants had to drag the mouse to alter the curvature via the segment height and click again once the line matched the perceived curvature of the motion. Thereafter, the next trial started.

In addition to these stimuli, there were two additional sets of stimuli according to the disturbance conditions. In the local disturbance condition, each dot had an additional motion component in a random direction.The speed of that component was chosen randomly from a Gaussian distribution with $1\, \sigma$ being equivalent to 4.2°/s or 60% of the pattern’s speed. This resulted in an average speed of the dots of 5.6°/s or 80% of the pattern’s speed. Within one trial the direction and speed of the motion components of each dot stayed the same across frames. In the global disturbance condition, all dots had an additional motion component uniformly either to the left or to the right. The speed of this additional global motion was 5.6°/s or 80% of the pattern’s movement. The three disturbance conditions were ordered in blocks with no disturbance first, then local disturbance, and then global disturbance. Between blocks participants could rest at will. The other conditions were all in random order. Altogether, there were 3 × 4 × 6 × 5 = 360 trials, three disturbance conditions (no disturbance, local disturbance, and global disturbance), four stimulus types (Rigid, Disc, Gaussian, and Constant), and six different end positions and five different curvatures.

The results were quantified by three performance measures. For each participant, stimulus type and disturbance condition we calculated the mean absolute error of direction (direction error), the mean absolute error of distance (distance error), and the proportion of trials in which a curvature was reported in the wrong direction (curvature error). The effect of the disturbance conditions on the performance was then further quantified in two ways. First, for each participant, the difference of the direction, distance, and curvature errors between the undisturbed condition and the local/global disturbance condition was calculated. Figure 3(q)–(t) shows the means of these differences over all participants with a 95% CI based on the variance across the 15 participants. Significance was tested with a repeated-measures t test. Due to a sample size of 15 all t-values greater than 2.145 were marked as significant. Second, for each single participant and each error type it was checked whether the error was greater in the global or the local disturbance condition. The distribution of participants more affected by either local or global disturbance was tested with an exact binomial test and distributions that were less than 0.05 likely to be caused by an equal strong effect of local and global disturbance were marked as significant.

Our choice to evaluate the curvature error categorically and not continuously was motivated by two observations. First, some of the participants responded categorically, drawing always a minuscule curvature, but the curvature always had the correct sign (100% correct for the Rigid stimulus). Perhaps this indicated some misunderstanding of the task instruction rather than a perceptual inability. Second, there are different ways to define a continuous error of curvature and a meaningful weighting of any chosen error norm is not trivial as the values are not normal-distributed. Therefore, a categorical analysis is more straightforward, not depended on further statistical analysis choices and better captures the response intentions of all participants.

For the speed discrimination task, two stimuli appeared in succession in random order. One was always the Rigid stimulus, in which the plate moved with a speed of 7.0°/s to either side in a straight line for a random duration between 0.75 and 1.33 s. The other stimulus was one of the nonrigid stimuli. In that stimulus, the vortex pattern moved with a speed of 3.3, 4.0, 4.8, 5.8, 7.0, 8.4, 10.2, 12.4, and 15.0°/s to either side in a straight line for a random duration between 0.75 and 1.33 s. Both stimuli of one trial moved toward the same side. Due to the random duration the end positions were not coupled to the speed of the stimulus. Participants had to indicate by button press which of the two successive stimuli appeared to move faster.

The speed discrimination experiment consisted of 3 × 9 × 2 × 5 = 270 trials. They included the three different nonrigid stimuli presented before or after the Rigid control stimulus, nine different speeds, and two directions of pattern movement. There were five repetitions. The nonrigid stimulus types were presented in three blocks in random order. Participants could rest in between. The other conditions were in random order within one block.

The speed perception data were fitted with psychometric curves of the form: $f(x)=g+(1-g-l)*(1-2^{(-(x/a)^{b}})$, where *a* is the 50% point, *b* the maximum slope, and *g* and *l* are guessing and lapse rate.

## Supplementary Material

pgac088_Supplemental_Files

## Data Availability

All data is included in the manuscript and its supplementary material.

## Previous Presentation

These results were previously presented at the Vision Sciences Society meeting 2021 (VSS 2021) on 2021 May 23.
