# Correction to: Perceived movement of nonrigid motion patterns

**DOI:** 10.1093/pnasnexus/pgad464

**Published:** 2024-01-30

This is a correction to: Krischan Koerfer, Markus Lappe, Perceived movement of nonrigid motion patterns, *PNAS Nexus*, Volume 1, Issue 3, July 2022, pgac088, https://doi.org/10.1093/pnasnexus/pgac088

During a retroactive audit conducted by *PNAS Nexus*, it was discovered that this paper was missing a statement acknowledging compliance with the *PNAS Nexus* Human and Animal Participants and Clinical Trials policy:

All participants were briefed about the experiments and provided written informed consent to participate in the study and for their data to be stored.

This error has been corrected in the original article.

