# Supplementary material for: Perceived movement of nonrigid motion patterns
Source: PNAS Nexus. 2022 Jun 22;1(3):pgac088. doi: 10.1093/pnasnexus/pgac088 (PMC9896959; doi:10.1093/pnasnexus/pgac088)
Supplement: pgac088_Supplemental_Files [file pgac088_supplemental_files.zip › About Figure S1.docx]

**Fig. S1. Curl fields are unaffected by global disturbance but degraded by the local disturbance.**

Two main findings of this study are that perception of the movement of all four motion patterns was possible and that local disturbance hampered perception more than global disturbance if the movement of motion patterns was the only cue. A plausible mechanism explaining these findings may utilize the curl of the motion field. Curl is a first order derivative of the motion field and a measure of the local rotation at a particular location in the field. Calculating it at all position in the field produces a field pattern of the distribution of curl. This figure shows the stimuli and the discrete numerical approximation of the resulting curl fields for the undisturbed (top rows), the globally disturbed (middle rows) and the locally disturbed (bottom rows) motion fields. Red indicates clockwise and blue counter-clockwise rotation. Saturation indicates strength. It can be clearly seen that adding global disturbance, which strongly distorts the pattern of the motion field (compare rows 1 and 3), does not affect the curl field (compare rows 2 and 4). Thus, a mechanism based on curl should be unaffected by the global disturbance, consistent with our data. Adding local disturbances (rows 5 and 6), on the other hand, degrades both, the motion field and its associated curl field. A mechanism based on curl should perform poorly, consistent with our data. A possible mechanism for perception of the movement of the vortices in our stimuli might consist in tracking of the extrema of the curl field over time.
