# Supplementary figures and images for: Perceived movement of nonrigid motion patterns
Source: PNAS Nexus. 2022 Jun 22;1(3):pgac088. doi: 10.1093/pnasnexus/pgac088 (PMC9896959; doi:10.1093/pnasnexus/pgac088)

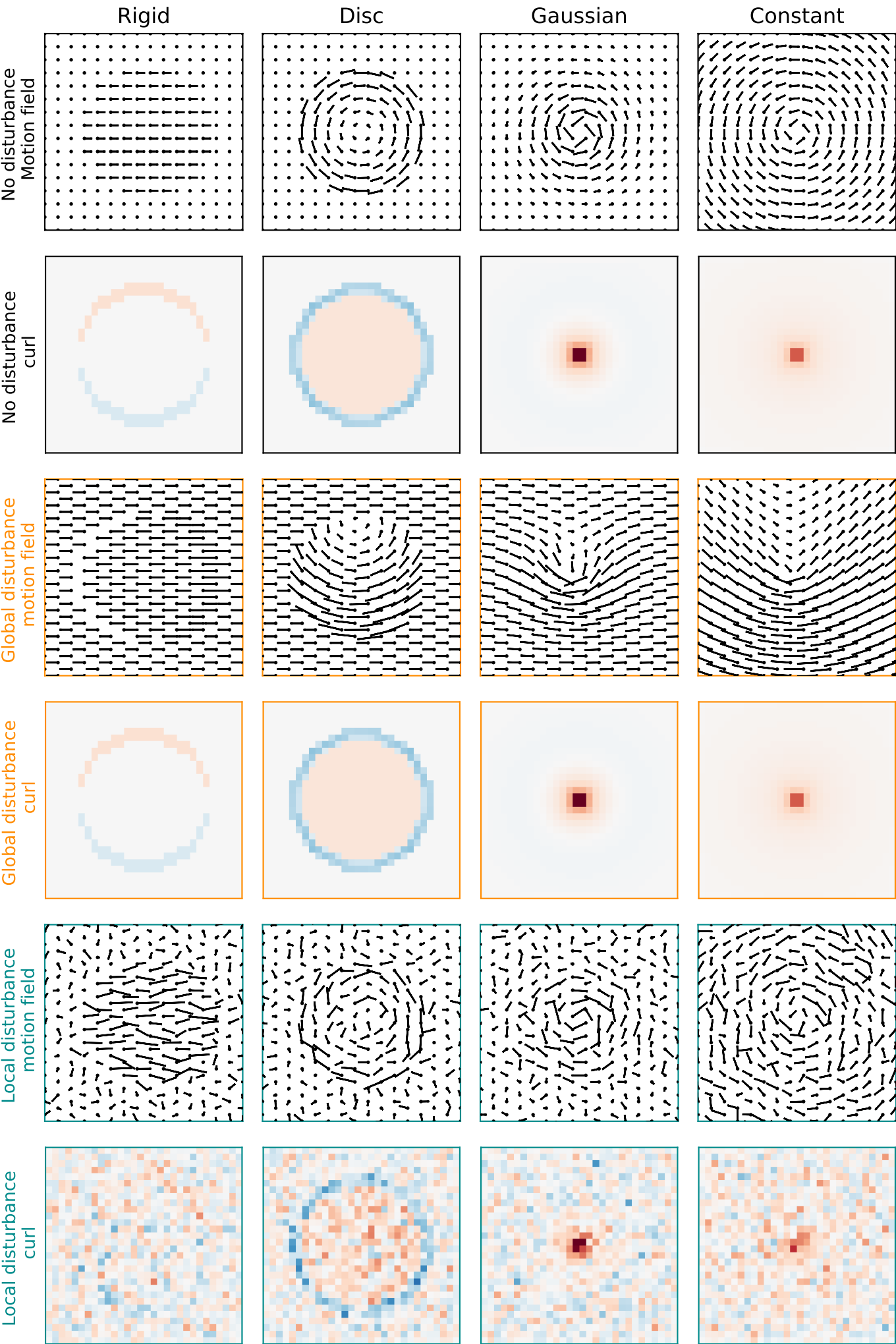

Supplement: pgac088_Supplemental_Files [file pgac088_supplemental_files.zip › FigureS1_curl_fields.pdf]

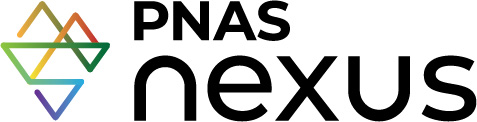

Supplement: pgac088_Supplemental_Files [file pgac088_supplemental_files.zip › PNASNEXUS-PNASNEXUS-2022-00268-T-s04.jpg]
